# Stability of Blueberry Extracellular Vesicles and Their Gene Regulation Effects in Intestinal Caco-2 Cells

**DOI:** 10.3390/biom13091412

**Published:** 2023-09-19

**Authors:** Yangfan Leng, Liubin Yang, Hangxin Zhu, Dongqin Li, Siyi Pan, Fang Yuan

**Affiliations:** 1 College of Food Science and Technology, Huazhong Agricultural University, Wuhan 430070, China; 593lyfmail.hzau.edu.cn@webmail.hzau.edu.cn (Y.L.); yangliubin@mail.hzau.edu.cn (L.Y.); zh_1@webmail.hzau.edu.cn (H.Z.); pansiyi@mail.hzau.edu.cn (S.P.); 2 National Key Laboratory of Crop Genetic Improvement, National Center of Plant Gene Research (Wuhan), Huazhong Agricultural University, Wuhan 430070, China; ldq@mail.hzau.edu.cn; 3 Hubei Key Laboratory of Fruit & Vegetable Processing & Quality Control, Huazhong Agricultural University, Wuhan 430070, China

**Keywords:** blueberry extracellular vesicles, stability, in vitro digestion, storage, LC-MS/MS

## Abstract

Plant extracellular vesicles (P-EVs) are considered promising functional food ingredients due to their various health benefits. In this study, blueberry extracellular vesicles (B-EVs) were collected and purified by size exclusion chromatography (SEC). The chemical compounds in B-EV extracts were analyzed by LC-MS/MS. In addition, the stability of B-EVs was evaluated during short- and long-term storage, heating, and in vitro digestion. The results showed that the B-EVs had a desirable particle size (88.2 ± 7.7 nm). Protein and total RNA concentrations were 582 ± 11.2 μg/mL and 15.4 μg/mL, respectively. The optimal storage temperatures for B-EVs were 4 °C and −80 °C for short- and long-term storage, respectively. Fluorescent labeling and qRT-PCR tests showed that B-EVs could be specifically internalized by Caco-2 cells, whereas virtually no cytotoxic or growth-inhibitory effects were observed. B-EVs down-regulated the expression levels of *IL-1β* and *IL-8* and up-regulated the expression levels of *NF-κβ* and *TLR5* in Caco-2 cells. Overall, the results proved that the intact structure of B-EVs could be preserved during food storage and processing conditions. B-EVs had the ability to reach the human intestine through oral delivery. Moreover, they could be absorbed by intestinal cells and affect human intestinal function.

## 1. Introduction

Plant extracellular vesicles (P-EVs) are membrane-enclosed vesicles with diameters of 50 nm–300 nm, containing bioactive molecules such as proteins, lipids, and RNAs. Plants excrete P-EVs for cell-to-cell communication, immune system regulation, and defense from pathogen invasions [1]. When the plants are consumed as food, P-EVs can be absorbed by the consumer’s intestinal macrophages and induce the expression of multiple cytokines in the host [2]. Among the cargos of P-EVs, microRNAs (miRNAs) have gained a lot of interest because they can regulate gene expression post-transcriptionally and exert multiple health benefits and functionalities [3]. For example, in ginger-derived extracellular vesicles (G-EVs), miRNA mdo-miR7267-3P induced interleukin-22 (IL-22) production to relieve the symptoms of colitis [4]. Another study reported that miR-CM1 derived from *Phellinus linteus* extracellular vesicles can inhibit the expression of *Mical2* to alleviate ultraviolet-induced skin aging [5]. In addition to miRNAs, proteins, lipids, and other chemicals were also reported to be functional components in P-EVs. The heat shock protein (HSPA8) in mulberry bark EVs can activate aryl hydrocarbon receptor (AHR)-mediated signaling in mice to prevent DSS-induced colitis [6]. Chen et al. [7] reported that the lipid of G-EVs can inhibit the assembly and activation of NLRP3 inflammasome to regulate inflammation. Additionally, strawberry-derived EVs can reduce reactive oxygen species (ROS) due to high vitamin C content [8]. These bioactive molecules can be encapsulated in P-EVs and manage to survive harsh conditions in the circulation of the host [9]. The P-EVs were able to maintain their structures when exposed to the ex vivo digestion process with only changes in size and charge [10]. When given orally, some P-EVs can also survive the digestion process, enter the colon area [10], and be absorbed by intestinal cells via endocytosis and further travel via the bloodstream [11]. However, since P-EVs are different from animal exosomes in many biological aspects, several basic problems are still unsolved. For example, protein markers need to be established and unified. PEVs contain multiple components, such as lipids, proteins, nucleic acids, and secondary metabolites, and their specific substances and mechanisms of action need to be further studied and explored.

Blueberry (*Vaccinium* spp.) has been reported to have multiple health benefits, such as oxidative stress regulation and anti-inflammation [12]. Blueberry extracellular vesicles (B-EVs) were reported to relieve oxidative stress by the positive modulation of HMOX1 and NRF1 in EA. hy926 cells [13]. Zhao et al. [14] also reported that B-EVs can decrease the expression levels of aspartate aminotransferase and alanine aminotransferase to improve insulin resistance and liver dysfunction and attenuate the accumulation of lipid droplets by inhibiting the expression of fatty acid synthase and acetyl-CoA carboxylase. In our recent study, we found that the miRNAs in B-EVs were predicted to target human genes involved in intestinal immune response [15], indicating that B-EVs might be used as a novel functional food ingredient to promote human intestinal health. Adhesion of B-EVs to the intestinal surface is an important property that confers immune system modulation. However, the stability of B-EVs during food processing, storage, and digestion has not been clearly demonstrated yet. Therefore, the objective of this study was to (i) determine the effect of storage conditions, heat treatments, and in vitro simulated gastrointestinal digestion on B-EVs’ stability; and (ii) investigate the internalization and gene regulation effects of B-EVs when contacting human intestinal cells.

## 2. Materials and Methods

### 2.1. Separation and Identification of B-EVs

Blueberries were purchased from a local market. Blueberries (250 g) with 1000 mL of phosphate-buffered saline solution (PBS) were well mixed using a household juicer, and then the mixture was used for extracting B-EVs according to a previously described method [15]. Briefly, the blueberry juice was centrifuged at 3000× *g* for 10 min, 8000× *g* for 30 min, and 10,000× *g* for 60 min at 4 °C. The supernatant was passed through a 0.22 µm filter and concentrated with a 10,000 MWCO membrane (Millipore, Shanghai, China). Finally, 10 mL of concentrated blueberry juice was added to a commercial qEV 10 column (Izon Science, Canterbury, New Zealand), and the 10 mL eluate was collected as fraction 1. Then 21.4 mL of PBS was loaded and collected as fraction 2. According to our previous research, the B-EVs were abundant in fraction 2 [15]. So fraction 2 was collected and concentrated to around 200 µL with a 10,000 MWCO membrane. The total protein concentration of B-EV isolates was determined using the BCA protein assay kit (Nanjing Jiancheng Bioengineering Institute, Nanjing, China).

The sizes of B-EVs were determined by dynamic light scattering (DLS) using a Zetasizer Nano-ZS 90 size analyzer (Malvern Instruments Ltd., Malvern, UK) at 25 °C [15]. The distribution of hydrodynamic diameter, D_h_, was determined by using the Stokes–Einstein relationship D_h_ = K_B_T/3πηD_0_, where K_B_ is the Boltzmann constant, T the absolute temperature, and η the solvent viscosity.

The morphology analysis of B-EVs was carried out by transmission electron microscopy (TEM). TEM analysis was performed following the published method with minor modifications [16]. The B-EVs were added to copper grids at room temperature. Then negative staining was applied to B-EVs for 30 s, using 1% aqueous phosphotungstic acid (PTA) solution (pH 6.4–7), which was positioned at room temperature until the PTA solution was dried completely. Measurements were carried out by using an FEI TECNAI 12 G2 Twin, operating at 80 kV and equipped with an electron energy loss filter and a slow-scan charge-coupled device camera (Hitachi, Tokyo, Japan).

The concentrated blueberry juice and B-EVs were analyzed using LC-MS/MS in positive ESI ionization mode. A Waters Vion IMS Qtof system was equipped with a Waters C18 column (XBridge^®^ C18 3.5 μm, 3.0 × 150 mm). The mobile phases were 0.1% formic acid in water (solvent A) and acetonitrile (solvent B). The gradient was 0–4 min to 100% B; 42–48 min to 5% B; and 48–51 min to 100% B. The mass spectrometer was operated with the following operating parameters: capillary voltage, 3.5 kV; column temperature, 25 °C; and velocity of flow, 300 μL/min. The source temperature was 120 °C and the desolvation temperature was 400 °C. The desolvation gas flow was 400 L/h. LC-MS/MS analysis follows similar steps as those outlined by Kyung et al. [17].

RNAs from B-EVs were extracted with an Eastep^®^ Super Total RNA Extraction Kit (Promega Co., Madison, WI, USA) according to the manufacturer’s instructions. Total RNA was collected and the concentration was measured by Qubit 3 Fluorometer (Invitrogen, Carlsbad, CA, USA).

### 2.2. Stability of Β-ΕVs under Heat Treatments

The extracted Β-ΕVs were subjected to two different heat treatments that may simulate pasteurization during food manufacturing. Half of the sample was heated at 60 °C for 30 min, while the other half was heated at 75 °C for 15 s. The stability of Β-ΕVs under heat treatments was evaluated according to changes in size, morphological features, and total protein, as described in 2.1. Each sample was assayed in triplicate.

### 2.3. Stability of Β-ΕVs during Storage

The Β-ΕV samples were divided into four groups with three replicates. Then the four groups were stored at −80 °C, −20 °C, 4 °C, and 25 °C for 7 d and 30 d, respectively. The stability of Β-ΕVs was evaluated by measuring their size and morphological features using the method described above.

### 2.4. Stability of Β-ΕVs in Simulated Gastrointestinal Tract Environment

In vitro stability tests were performed according to the published method with minor modifications [10]. Totals of 1.34 μL of 18.5% (*w*/*v*) HCl (pH 2.0) and 24 μL of pepsin solution (80 mg/mL in 0.1 mol/L HCl, pH 2.0) were added to 1 mL (1 mg/mL) of Β-ΕVs in PBS, and the mixture was incubated at 37 °C for 0.5 h (stomach-like conditions). Then, 80 μL of a mixture containing 24 mg/mL of bile extract and 4 mg/mL of pancreatin in 0.1 N NaHCO_3_ was added. The pH was adjusted to 6.5 with 1 N NaHCO_3_ and incubated for an additional 0.5 h at 37 °C (intestine-like conditions). The stability of Β-ΕVs was evaluated by measuring their size and morphological features using the method described above.

### 2.5. Caco-2 Cell Culture

The human Caco-2 intestinal epithelial cell line was purchased from Procell (Wuhan, China). The culture medium contained 89% Dulbecco’s Modified Eagle Medium (DMEM, GIBCO, Shanghai, China), 10% fetal bovine serum (FBS, GIBCO, China), and 1% penicillin–streptomycin (Biosharp, Hefei, China). Cells were cultured in a T75 culture flask (Thermo Scientific™, Shanghai, China) and incubated in a carbon dioxide incubator at 37 °C and 5% CO_2_. An inverted microscope (Nikon Ti-S, Tokyo, Japan) was used to observe cell adhesion and growth. The culture medium was changed every 24 h. Cell transmission follows the same steps as described in Bruno et al. [18].

### 2.6. Cytotoxicity Assay

A cytotoxicity assay was performed according to the published method with minor modifications [18]. Caco-2 cells were seeded in three 96-well plates at a density of 1 × 10^4^ cells/well with 100 μL of culture medium and incubated for 4~6 h to promote cell adhesion to the wall. Β-ΕV concentration was expressed as the concentration of protein in the extract. Additionally, the concentration gradient was determined after a careful review of previous publications [8,18,19]. Adherent cells were then treated with 10 μL of culture medium mixed with different concentrations of Β-ΕVs (0, 5, 10, 20 μg/mL, with 7 repetitions per group) and incubated for 12 h, 24 h, and 36 h. At each time point, the Β-ΕV-containing medium was removed and cells were thoroughly rinsed twice with PBS. Then, 100 μL of fresh, non-supplemented DMEM and 10 μL of CCK8 (Beyotime, Shanghai, China) were added in each well. After 1 h incubation, absorption was measured by a Multskan GO microplate reader (Thermo, Waltham, MA, USA) at a wavelength of 450 nm. Student’s *t*-test was used to analyze cell cytotoxicity at different times and concentrations.

### 2.7. Preparation of Caco-2 Monolayers

Caco-2 cells were seeded onto polycarbonate filters inside Transwell cell culture chambers (Servicebio, Wuhan, China) at a density of 2 × 10^5^ cells/cm^2^. The culture medium was added to the chamber (0.5 mL in the insert and 1.5 mL in the well). The transepithelial electric resistance (TEER) of the monolayers was checked using an electrical resistance system (Millicell, Shanghai, China). The culture medium changing and transmission followed the same protocols as those described by Saliba et al. [20] A TEER value higher than 500 Ohm·cm^2^ indicates that the cells have fully differentiated [21].

### 2.8. Β-ΕV Labeling

Fluorescent labeling of Β-ΕVs was carried out using the pKH26 Fluorescent Cell Ligation Kit (Solarbio, Beijing, China) according to the manufacturer’s instructions. Briefly, equal volumes of samples were mixed with dye and incubated at 37 °C for 30 min. Subsequently, 1 × PBS was added and the sample was centrifuged for 1 h at 120,000× *g*, repeating three times to remove the unbound-free dye. Labeled Β-ΕVs were resuspended in DMEM for further experiments.

### 2.9. In Vitro Internalization of Β-ΕVs by Caco-2 Cells

Labeled Β-ΕVs were incubated with fully differentiated Caco-2 cells for 6 h, 12 h, and 24 h. After incubation, cells were fixed with 4% paraformaldehyde (PFA) for 10 min. Then DAPI (Beyotime, Shanghai, China) and DIO (Beyotime, Shanghai, China) were added to label the cell nucleus and cell membranes, respectively. Finally, cells were coverslip-mounted and a confocal laser scanning microscope (Olympus FV3000, Tokyo, Japan) was used to capture the images. The excitation wavelengths of DIO, PKH26, and DAPI were 484 nm, 340 nm, and 551 nm, respectively. Additionally, to explore the mechanisms associated with Β-ΕV absorption, the endocytosis inhibitor (Chlorpromazine) was preincubated with Caco-2 cells for 1 h. The labeled Β-ΕVs were added to fully differentiated Caco-2 cells and incubated for another 24 h at 37 °C. Caco-2 cells were seeded onto 12-well plates and were incubated with Β-ΕVs for 24 h. Cells without Β-ΕVs were used as a negative control.

### 2.10. Quantitative Real-Time Polymerase Chain Reaction (qRT-PCR)

After incubation, the total RNA of Caco-2 cells was extracted using the phenol–chloroform method. First-strand cDNA was synthesized by using the SweScript All-in-One First-Strand cDNA Synthesis SuperMix Kit (One-Step gDNA Remover) (Wuhan Servicebio Technology Co., Ltd., Wuhan, China). The first-strand cDNA mix was prepared by combining 4 µL of 5 × SweScript All-in-One SuperMix Kit for qPCR, 1 µg of total RNA, and 1 µL of gDNA remover, and then nuclease-free water was added for a final volume of 20 µL. The GAPDH gene was used as a control and specific primers were designed based on cDNA sequences (Table S1). Real-time PCR was performed with the following program: 95 °C for 30 s, followed by 40 cycles at 95 °C for 15 s, 60 °C for 10 s, and 72 min for 3. Relative gene expression was calculated using the Livak and Schmittgen 2^−ΔΔCt^ method [22], normalized with the reference gene GAPDH. QPCR amplifications were performed in triplicate for each sample. The data were examined by *t*-test.

### 2.11. Statistical Analysis

Statistical analysis was performed using SPSS 22.0 (SPSS, Chicago, IL, USA). All data were representative of three independent experiments and statistical differences were considered significant at * *p* < 0.05 or ** *p* < 0.01. Image processing was performed using Origin Pro 2019b (Origin Lab Corporation, Northampton, MA, USA).

## 3. Results

### 3.1. Characterizations of B-EVs Obtained via SEC Methods

**Figure 1:**
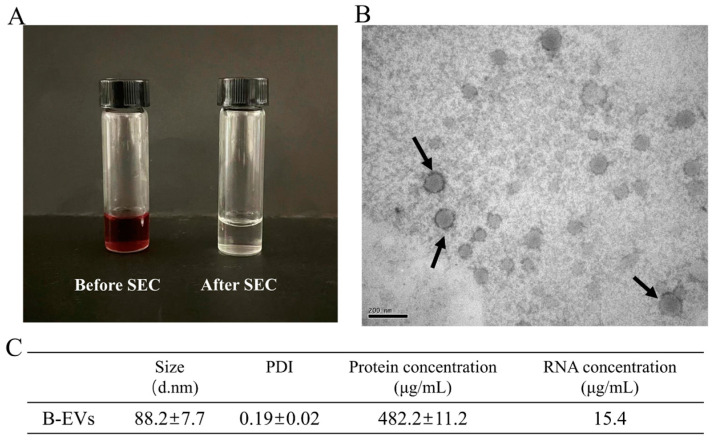
Characterization of B-EVs extracted by SEC. (**A**) A picture of centrifuged blueberry juice (before SEC) and B-EVs. (**B**) TEM image of B-EVs isolated via SEC. (**C**) The size, total protein concentration, and RNA concentration of B-EVs.

Size exclusion chromatography (SEC) is becoming a widely used technique to extract and purify P-EVs using simple steps. In this study, SEC was used to extract the EVs derived from blueberries. SEC performed well in removing the anthocyanins in the blueberry, as the color of the solution changed from purple to transparent after SEC extraction (Figure 1A). The results showed that B-EVs extracted by SEC had a round shape with a clear membrane structure (Figure 1B), desirable particle size (88.2 ± 7.7 nm), and a low PDI (0.19 ± 0.02) (Figure 1C and Table S2). The B-EV extract had 482.2 ± 11.2 μg/mL of protein and 15.4 μg/mL of RNA. Compared with other studies, the SEC method was more effective in removing protein contaminants of P-EVs (Table S3). Additionally, LC-MS/MS was used to compare the compound in the centrifuged blueberry juice (before SEC) and in the B-EVs extract. As presented in Table 1, before SEC, the centrifuged blueberry juice was enriched with polyphenols, flavonoids, and anthocyanins (Figure S1). However, only 6-O-Malonylgenistin was detected in the B-EV extract purified by SEC, and the peak area was reduced by about 50%.

### 3.2. Stability of Β-ΕVs under Heat Treatments

**Figure 2:**
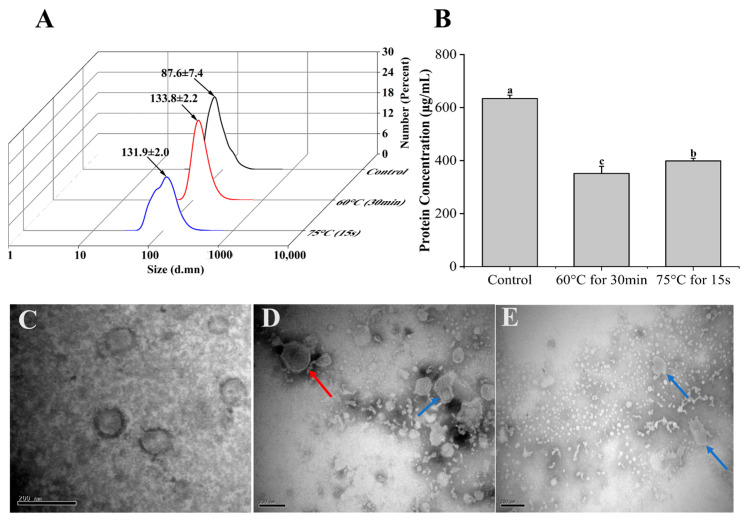
Stability of Β-ΕVs in a simulated food processing environment. (**A**) Size distribution of Β-ΕVs from two heat treatments (60 °C for 30 min; 75 °C for 15 s). The black arrows represent the mean of the main peak of Β-ΕVs; (**B**) protein concentration of Β-ΕVs after heat treatments. Different letters in the column chart represent significantly (Tukey HSD, *p* < 0.05) different in means; (**C**) TEM image of freshly extracted Β-ΕVs; (**D**) TEM image of Β-ΕVs heated at 60 °C for 30 min; (**E**) TEM image of Β-ΕVs heated at 75 °C for 15 s. The red arrows represent aggregated Β-ΕVs and the blue arrows show morphologically changed Β-ΕVs. Data are represented as mean ± standard error. scale bar: 20 nm.

The stability of Β-ΕVs was first evaluated under two different heat treatment conditions (60 °C for 30 min and 75 °C for 15 s) that mimic traditional pasteurization. After heat treatments, the size distribution, morphology, and protein content of Β-ΕVs were measured and compared with non-treated Β-ΕVs (Control) (Figure 2A). Transmission electron microscopy (TEM) showed that the Β-ΕVs were cup-shaped with a clear membrane structure. Dynamic light scattering (DLS) showed that the freshly extracted Β-ΕVs had an asymmetric size distribution of 50 nm to 220 nm, with an average size of 87.6 nm. After heating at 60 °C for 30 min and at 75 °C for 15 s, the average diameter of Β-ΕVs increased 35% and 33%, respectively. Heat treatments lowered the total protein content in the Β-ΕV extract. Both heat treatments changed the Β-ΕVs’ morphology, as part of the flat cup-shaped vesicles turned into polygon-shaped vesicles, and more aggregates were observed.

### 3.3. Stability of Β-ΕVs during Storage

**Figure 3:**
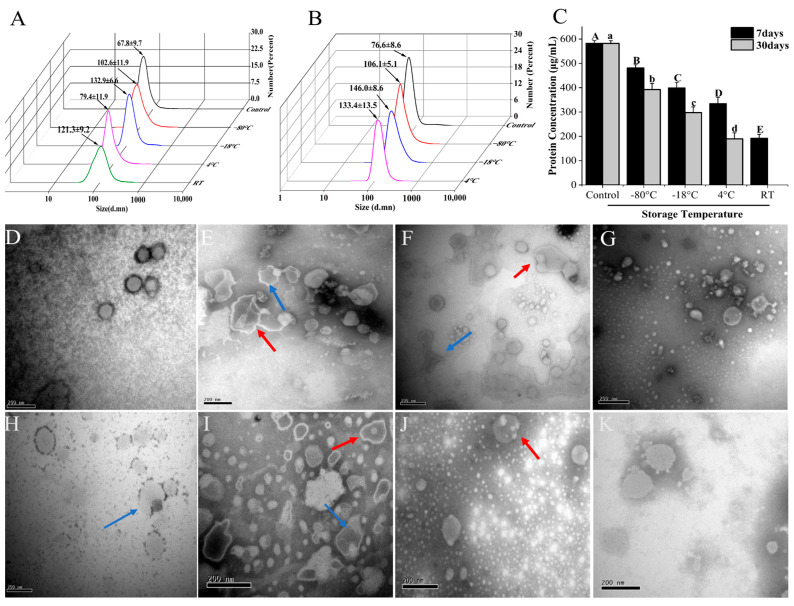
Stability of Β-ΕVs under various storage temperatures. (**A**) Particle sizes of Β-ΕVs stored at −80 °C, −18 °C, 4 °C, and 25 °C for 7 days; (**B**) particle sizes of Β-ΕVs stored at −80 °C, −18 °C, and 4 °C for 30 days; (**C**) protein concentration of Β-ΕVs under various storage temperatures. Different letters in the column chart represent significantly (Tukey HSD, *p* < 0.05) different in means; TEM images of Β-ΕVs stored for 7 days: control (**D**), −80 °C (**E**), −18 °C (**F**), 4 °C (**G**), and 25 °C (**H**); TEM images of Β-ΕVs stored for 30 days: −80 °C (**I**), −18 °C (**J**), 4 °C (**K**). The red arrows represent aggregated Β-ΕVs and the blue arrows show morphologically changed Β-ΕVs. Data are represented as mean ± standard error. scale bar: 20 nm.

Β-ΕVs were stored at four different temperatures (−80 °C, −20 °C, 4 °C, and 25 °C) for 7 days and 30 days, respectively (Figure 3). However, since the storage of Β-ΕVs at 25 °C for 30 days led to severe microbial growth, the data were discarded. Freezing storage is the most commonly used storage condition for P-ΕVs, but our results showed that freezing conditions resulted in morphological changes to Β-ΕVs. Β-ΕVs stored at −80 °C and −20 °C for 7 days were larger in size (they increased by 34% and 49%, respectively) compared to freshly isolated Β-ΕVs, and some larger nanovesicle aggregates of up to 300 nm in diameter were generated after freezing for 7 days. TEM observation also showed that after freezing, Β-ΕVs were becoming larger and more aggregated, and had more multi-lamellar membrane layers (Figure 3E,F). For the long-term storage (30 days), DLS data also showed that the size of Β-ΕVs increased. After 30 days of storage at −80 °C and −20 °C, the average diameter increased 28% and 48%, respectively, compared to the control (Figure 3B,I). The protein content decreased in comparison to the control, regardless of short-term or long-term preservation (Figure 3C).

Since refrigerator and room temperature storage are also common for fruits and fruit juice, we tested the stability of Β-ΕVs at 4 °C and 25 °C. The size and shape of Β-ΕVs stored at 4 °C for 7 days were similar in comparison to the control (Figure 3G). But after 30 days, the Β-ΕVs showed full or partial degradation (Figure 3K). The size of Β-ΕVs increased 44% when stored at 25 °C for 7 days. It was observed that the membrane structure of some Β-ΕVs became discontinuous (Figure 3H).

### 3.4. Stability of Β-ΕVs in In Vitro Digestion Conditions

**Figure 4:**
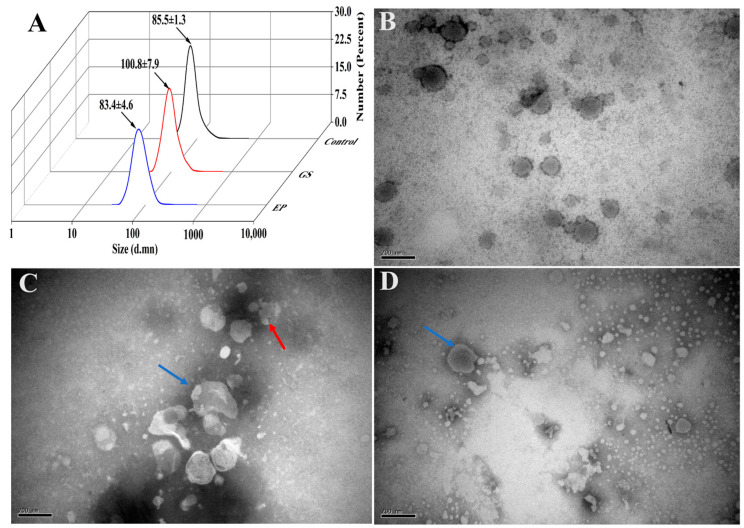
Stability of Β-ΕVs in a simulated gastrointestinal tract environment. (**A**) The sizes of Β-ΕVs before treatment (control), after treatment with GS (GS), and after treatment with GS followed by an EP solution (EP); (**B**) TEM image of freshly extracted Β-ΕVs; (**C**) TEM image of Β-ΕVs in simulated GS solution; (**D**) TEM image of Β-ΕVs in simulated EP. The red arrows represent aggregated Β-ΕVs and the blue arrows show morphologically changed Β-ΕVs. scale bar: 20 nm.

The average dimension of Β-ΕVs incubated in the gastric (GS) solution enlarged from 85.5 nm to 100.8 nm (Figure 4A). But Β-ΕVs in the following enteric phase (EP) solution showed a significant dimensional decrease compared with Β-ΕVs in the GS solution (from 100.8 ± 7.9 nm to 83.4 ± 4.6 nm). The total protein content had a dramatic increase due to the addition of enzymes in simulated gastrointestinal processing. TEM showed that the Β-ΕVs aggregated after treatment with the GS solution, which was highly correlated with the DLS result with respect to the size of Β-ΕVs increasing (Figure 4C). TEM results also showed that the sizes and shapes of most Β-ΕVs were preserved. The membranes of Β-ΕVs were degraded into smaller pieces when Β-ΕVs were incubated in the EP solution (Figure 4D).

### 3.5. Cytotoxicity of Β-ΕVs against Caco-2 Cells

**Figure 5:**
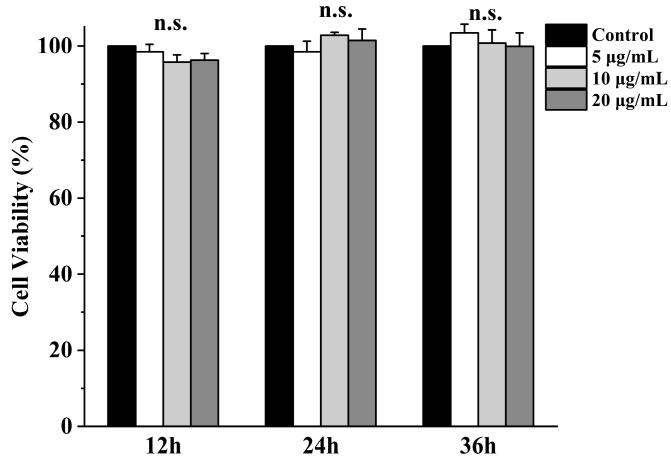
Viability of Caco-2 cells after Β-ΕV treatment. Cell viability was assessed by MTT assay at 12 h, 24 h, and 36 h after incubation with 5, 10, and 20 μg/mL Β-ΕVs, respectively. “n.s.” means no significance.

Β-ΕVs were co-cultured with Caco-2 cells for 12 h, 24 h, and 36 h, respectively. No statistical differences were observed between different Β-ΕV treatments and the control group, indicating that Β-ΕVs were not cytotoxic to Caco-2 cells (Figure 5).

### 3.6. Uptake and Transepithelial Transport of Β-ΕVs across Intestinal Epithelium In Vitro

**Figure 6:**
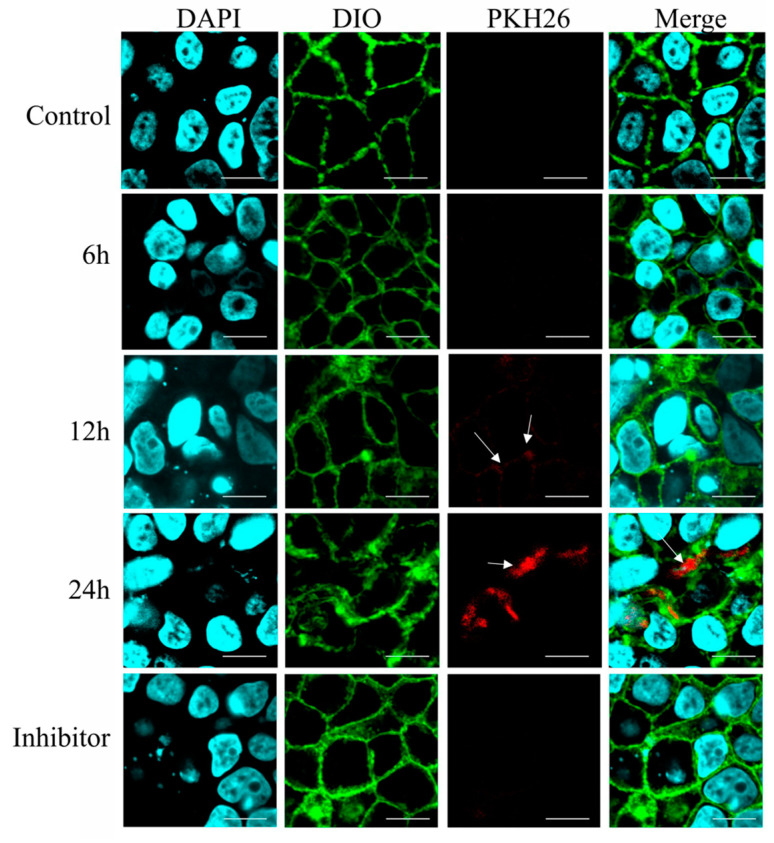
Fluorescence microscopy images of Caco-2 cells incubated with the PKH26-labeled Β-ΕVs for 6 h, 12 h, and 24 h. Inhibitor: Caco-2 cells were pretreated with chlorpromazine for 30 min and then incubated with Β-ΕVs. Caco-2 cell nuclei were labeled with DAPI (blue); the Caco-2 cell membrane was labeled with DIO (green); and Β-ΕVs were labeled with PKH26 (red). The white arrows showed the PKH26-labeled Β-ΕVs.

To monitor the dynamic process of Β-ΕV absorption, Caco-2 cells were exposed to PKH26-labeled Β-ΕVs with red fluorescence for 6 h, 12 h, and 24 h. As shown in Figure 5, there was no red fluorescence inside the cells at 6 h. After 12 h of incubation, a few red spots were distributed between the nucleus (blue) and the cell membrane (green) of the Caco-2 cells, indicating that the Β-ΕVs began to be internalized. And after 24 h, a large number of red spots were concentrated around the blue nucleus of the Caco-2 cells, implying that Β-ΕVs had completely entered Caco-2 cells. Confocal analysis showed that when the Caco-2 cells were pre-treated with chlorpromazine, no red vesicles were observed in the Caco-2 cells (Figure 6).

### 3.7. Effect of Β-ΕVs on Inflammatory Response in the Intestinal Epithelium Cells

**Figure 7:**
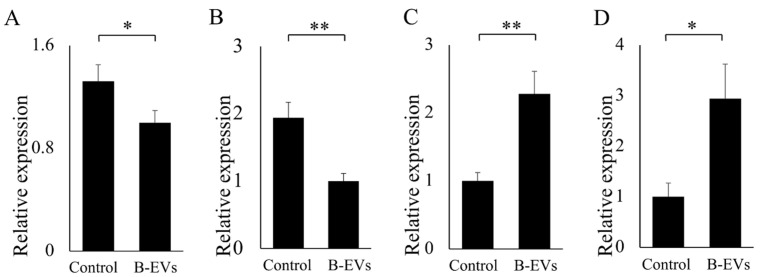
Effect of Β-ΕVs on the relative expression of genes related to inflammation. (**A**) *IL-1β*; (**B**) *ΙL-8*; (**C**) *TLR5*; (**D**) *NF-κβ.* Statistical significance is indicated as * for *p <* 0.05 and ** for *p <* 0.01.

According to the confocal results, Β-ΕVs were completely internalized after 24 h of co-culturing. So we speculated that after 24 h, the effect of Β-ΕVs on inflammatory response would be obvious. Hence, Β-ΕVs and Caco-2 cells were co-cultured for 24 h and the changes in gene expression were measured using quantitative real-time PCR (qRT-PCR). Ten inflammatory-related genes were selected according to the literature and predicted target genes for miRNA of Β-ΕVs [15]. Four out of the ten genes had a significant expression change. The results showed that Β-ΕVs down-regulated the gene expression levels of interleukin 1β (*IL-1β*) and interleukin 8 (*IL-8*), whereas the nuclear factor kappa-light-chain-enhancer of activated B cells (*NF-κβ*) and toll-like receptor 5 (*TLR5*) gene expression levels were up-regulated compared to the control group (Figure 7).

## 4. Discussion

Multiple studies have shown that edible P-EVs have various health benefits for consumers. However, the stability of P-EVs under heat treatments and storage conditions is important to evaluate in terms of their suitability to serve as novel functional food ingredients. Our results showed that B-EVs could maintain their structural integrity at pasteurization temperatures, speculating that some bioactive components of B-EVs, such as miRNA and proteins, were able to access the human intestine because of the encapsulated capability of B-EVs. Our result was similar to the report of Li et al. [23], which suggested that EVs could be found in traditional Chinese medicine boiled with water for 30 min, and lipids, small molecule metabolites, proteins, and sRNAs could also be detected after boiling. EVs were also found in cooked pork [24] and roasted hot coffee beverages [25], indicating that, although they were from different sources, EVs generally had a good resistance to heat treatments. Some studies suggested that the interaction between endogenous miRNAs and exosomes might promote the stability of miRNAs during heat treatment [25,26]. However, other studies suggested that whether the stability of miRNAs in cooked or processed food was solely due to encapsulation by P-EVs still needed further investigation [11], since many sRNAs in *Arabidopsis* leaf EVs were more likely to be associated with proteins outside the membranes instead of being fully encapsulated inside EVs [27].

Storage is a crucial factor that influences the physical properties and functionality of P-EVs. Surprisingly little is known about which condition is the most suitable for the storage of P-EVs. Although the stability of extracted EVs from mammals has been examined by accumulated studies, there remains some controversy about the influence of different storage temperatures on EVs. The majority of studies have suggested that mammal-derived EVs be stored at −80 °C [28,29]. Additionally, it seems to be commonly recognized that P-EVs should also be preserved at −80 °C [30]. In this study, we observed the particle size increase and morphological changes of B-EVs during storage at different temperatures. Although −80 °C storage could maintain the particle size of B-EVs, freezing resulted in more “vesicles in vesicle” shaped B-EVs, with some vesicles being dense and others seeming to be empty. This phenomenon could be explained, in part, by the phospholipid bilayer membrane of B-EVs possibly being damaged by ice crystals during freezing, and these crystals producing the de-mixing of biological surfactants, resulting in a fusion between miscible membranes during dehydration and shaping of the multilamellar vesicles [31]. When compared to −80 °C, B-EVs stored at 4 °C for 7 days were still round shaped with a clear membrane structure. Considering storage at −80 °C is prohibitively costly, 4 °C storage of B-EVs is an alternative way to preserve B-EVs in the short term, which is consistent with the results of previous reports that HEK 293 T cell-derived EVs stored at 4 °C or 37 °C for 24 h were better than those stored at freezing temperatures [32]. However, as the storage time increased to 30 days, B-EVs started to swell, and the membrane degraded quickly as the temperature increased. So for long-term storage, it is still recommended to store B-EVs at −80 °C. Additionally, it is worth mentioning that, in our experience, a repeated freeze–thaw cycle may cause significant damage to B-EVs because of the fragility of phosphatidylserine on the lipid bilayer of B-EVs when exposed to ice crystals [33].

The stability of B-EVs in human digestive systems should be clearly demonstrated before B-EVs could be utilized as functional food ingredients. Our study showed that the microstructures of some B-EVs were altered after incubation in simulated gastrointestinal fluids. Extensive particle aggregation was observed in simulated gastric fluids, and some of the large aggregates formed in the GS solution then broke down into smaller clumps after exposure to the EP solution. This might be caused by partial hydrolysis of B-EVs by enzymes or by changes in electrostatic interactions caused by alterations in ionic strength and pH. The stability of P-EVs from other plant species has been tested in the human digestive system, but the results are still subject to debate. Reports of P-EV size changes during the digestive process were prevalent. Similar to our observation, the size of oat-derived EVs increased in a stomach-like solution and then decreased in a small-intestine-like solution [34]. However, another study suggested that the sizes of citrus-derived EVs were not changed after passing through the gastric solution, but increased in the intestinal solution. [18] The size of ginger-derived EVs was reduced slightly in a simulated gastrointestinal tract environment [10]. In contrast, other research showed that tartary buckwheat-derived EVs had no obvious change in their particle size and zeta potential during passage through a simulated gastrointestinal tract [35]. These results indicated that EVs from different plant species might have different abilities to resist gastrointestinal digestion. In addition, other particles in P-EV extracts, such as proteins and cell debris, could also affect the size measurement results. So different extraction and purification methods for P-EVs should also be considered.

The Caco-2 cell line is derived from human intestinal epithelium and is a widely accepted in vitro model for studying cytotoxicity and delivery [20]. Our results showed that B-EVs were non-toxic and harmless to Caco-2 cells. The result is consistent with many studies supporting that EVs derived from edible plants are nontoxic. For example, the concentration gradient of strawberry EVs was from 2 to 9 μg/mL, confirming that strawberry EVs did not exert any significant toxic effect on the adipose-derived mesenchymal stem cells [8]. Citrus EVs and pomegranate EVs did not significantly alter the cell viability of Caco-2 cells [19]. The cytotoxicity of plant-derived EVs was low, probably because P-EVs were composed of natural origin cellular components and derived from plants. Our results also suggested that B-EVs could be easily internalized by Caco-2 cells via endocytosis and distributed throughout the host cytoplasm after endocytosis. Similar results in terms of intracellular distribution were also reported in other research [18,36]. After being internalized by Caco-2 cells, B-EVs modulated the expression of important genes related to inflammatory pathways in the Caco-2 cells, such as *IL-1β, IL-8, TLR5*, and *NF-κβ*, to affect intestinal inflammation [37]. Among them, *NF-κβ* was a central player in the signaling cascades mediating inflammation. Another study reported that the production of *NF-κβ*, cytokines, and chemokines via the triggering of toll-like receptor 4 (*TLR4*) could activate endogenous immunity, which is beneficial to the organism to a certain extent [38]. Previous studies suggested that EVs were effective vehicles for transferring plant-derived miRNAs to mammalian cells. Additionally, plant miRNAs have shown targeting effects on mammalian gene expression both in vivo and in vitro [18,31]. Our previous research also found that *TLR 5* was the predicted target gene for the miR-466i of B-EVs. In this study, we inferred that B-EVs were effective delivery systems, and the abundant microRNA within EVs may play regulatory roles in the expression of immune-related genes. Incontestably, there were still a few unavoidable contaminants in the B-EV extracts, such as proteins and anthocyanins, which might also play a regulatory role. P-EVs utilize proteins to carry out diverse cellular functions and facilitate intercellular communication by transferring their cargo to recipient cells, resulting in antioxidant stress reduction and anti-inflammatory activation [39,40]. Further research is needed to comprehensively identify the wide range of P-EV proteins involved in their biological and pharmacological activities. Our results indicated that B-EVs regulated some genes in Caco-2 cells, but the downstream bioprocess of these changes is not clear. More ex vivo studies are still needed to explore the role of B-EVs as a food component in regulating human intestinal health.

## 5. Conclusions

This study proposed effective storage conditions for B-EVs: 4 °C for short-term storage and −80 °C for long-term storage. The 4 °C storage could prevent the ice crystals from damaging the phospholipid bilayer membrane of B-EVs, while −80 °C could slow down the degradation rate. B-EVs had certain stability in simulated gastrointestinal and processing environments. In addition, B-EVs were not cytotoxic and could be easily internalized by intestinal epithelium cells, regulating the host inflammation response. These results provided a theoretical basis for blueberry-derived EVs as a novel functional food ingredient. It is worth mentioning that there is still a lack of a one-size-fits-all solution for the storage of P-EVs. Further research is still needed to obtain B-EVs with higher efficiency and higher purity, which could be helpful in verifying the effect of B-EVs in vivo.

## Figures and Tables

**Table 1:** Compounds identified in centrifuged Blueberry juice (before SEC) and in the B-EV extract via LC-MS/MS.

No.	Compounds	Molecular Formula	RT (min)	Precursor (*m*/*z*)	MS/MS (*m*/*z*)	Response (Centrifuged Blueberry Juice)	Response (B-EV Extract)
1	3-O-Methylgallic acid	C_8_H_8_O_5_	5.16	185.0429	142.00346	942.3 ± 54.5	ND
2	Coumarin	C_9_H_6_O_2_	5.34	147.044	102.96836	2783.0 ± 334.0	ND
3	Malvidin 3-O-β-D-galactoside	C_23_H_25_O_12_	48.15	493.1339	332.08495	32,490.7 ± 4927.0	ND
4	Isopeonidin 3-O-arabinoside	C_21_H_21_O_10_	49.48	433.1124	270.02277	2921.3 ± 1644.1	ND
5	Malvidin 3-arabinosid	C_22_H_23_O_11_	49.59	463.1234	301.06164	17,968.0 ± 6999.2	ND
6	5,6,7,3′,4′-Pentahydroxyisoflavone	C_15_H_10_O_7_	50.35	303.0492	257.04602	10,522.0 ± 2943.6	ND
7	Petunidin-glucoside	C_22_H_23_O_12_	50.37	479.1192	317.06595	1748 ± 301	ND
8	Luteolin	C_15_H_10_O_6_	50.47	287.0546	133.12206	950.7 ± 133.4	ND
9	6-O-Malonylgenistin	C_24_H_22_O_13_	51.18	519.1139	270.44986	8657.1 ± 1025.8	4340 ± 660.1

## Data Availability

Data will be made available on request.

## Supplementary Materials

The following supporting information can be downloaded at: https://www.mdpi.com/article/10.3390/biom13091412/s1. Table S1. Primer Sequences Used for Quantitative RT-PCR. Table S2. Polydispersity Indexes of B-EVs under various conditions. Table S3. Comparison of the protein concentrations in EVs isolated from different sources. Figure S1. (A) The total ion chromatogram of centrifuged blueberry juice (before SEC). (B) The total ion chromatogram of B-EVs. Mass spectrogram of 3-O-Methylgallic acid (C), Coumarin (D), Malvidin 3-O-β-D-galactoside (E), Isopeonidin 3-O-arabinoside (F), Malvidin 3-arabinosid (G), 5,6,7,3′,4′-Pentahydroxyisoflavone (H), Petunidin- glucoside (I), Luteolin (J), 6-O-Malonylgenistin (K). References [41,42,43] are cited in Supplementary Materials.
