# Supplementary material for: Stability of Blueberry Extracellular Vesicles and Their Gene Regulation Effects in Intestinal Caco-2 Cells
Source: Biomolecules. 2023 Sep 19;13(9):1412. doi: 10.3390/biom13091412 (PMC10526224; doi:10.3390/biom13091412)
Supplement: Supplementary file 1 [file biomolecules-13-01412-s001.zip › biomolecules-2622448-supplementary.pdf]

Stability of Blueberry Extracellular Vesicles and Their Gene Regulation Effects in Intestinal Caoco-2 Cells

Table S1. Primer Sequences Used for Quantitative RT-PCR

Primer	Sequence (5'to3')
IL-10 F	tct ccg aga tgc ctt cag cag a
IL-10 R	tca gac aag gct tgg caa ccc a
IL2RA F	gag act tcc tgc ctc gtc aca a
IL2RA R	gat cag cag gaa aac aca gcc g
TLR5 F	cct tac agc gaa cct cat cca c
TLR5 R	tcc act aca gga gga gaa gcg a
FOXP3 F	ggc aca atg tct cct cca gag a
FOXP3 R	cag atg aag cct tgg tca gtg c
TGFB2 F	aag aag cgt gct ttg gat gcg g
TGFB2 R	atg ctc cag cac aga agt tgg c
IL-6 F	aga cag cca ctc acc tct tca g
IL-6 R	ttc tgc cag tgc ctc ttt gct g
IL-8 F	ctg gcc gtg gct ctc ttg
IL-8 R	ggg tgg aaa ggt ttg gag tat g
TNF-α F	tca acc tcc tct ctg cca tc
TNF-α R	cca aag tag acc tgc cca ga
IL-1β F	aca gat gaa gtg ctc ctt cca
IL-1β R	gtc gga gat tcg tag ctg gat
NF-κβ F	gga ttt cgt ttc cgt tat gta tg
NF-κβ R	tcc ttg ggt cca gca gtt a

Table S2. Polydispersity Indexes of B-EVs under various conditions

	Treatment	PDI
Control	Fresh	0.188±0.01
7 days	-80 °C	0.231±0.04
	-20°C	0.345±0.06
	4°C	0.215±0.03
	25°C	0.291±0.03
30days	-80 °C	0.26±0.01
	-20°C	0.407±0.06
	4°C	0.255±0.04
Heat treatments	60°C for 30min	0.306±0.05
	75°C for 15s	0.322±0.01
In vitro digestion	GS	0.316±0.04
	EP	0.298±0.02

Table S3. Comparison of the protein concentrations in EVs isolated from different sources.

Species	Extraction method	Protein concentration（μg/mL）
Ginger[36]	Ultracentrifugation	2391.43± 750
Periplaneta americana L.[41]	Ultracentrifugation	2330±440
Citrus[18]	Ultracentrifugation	1000
Blueberry[42]	Ultracentrifugation	5599.96±132.62
Blueberry	Size exclusion chromatography	482.2±11.2
Phytophthora capsica[43]	Ultracentrifugation	500

A

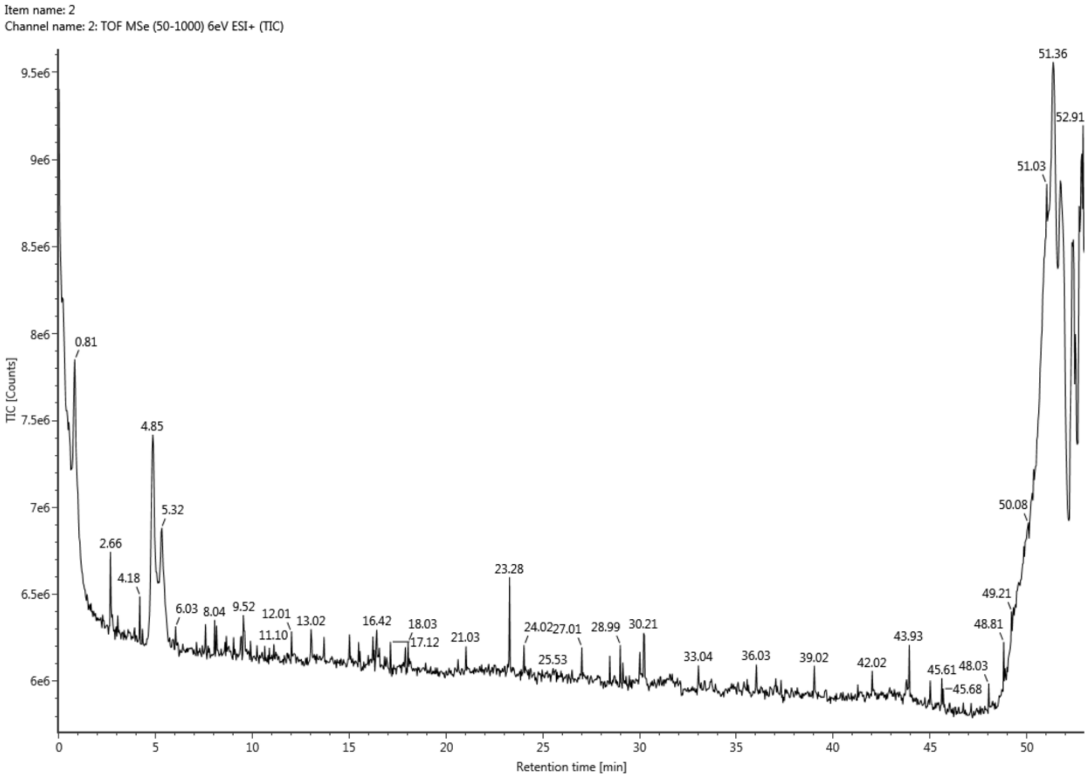
Item name: 2
Channel name: 2: TOF MSe (50-1000) 6eV ESI+ (TIC)
TIC [Counts]
Retention time [min]
0.81
2.66
4.18
4.85
5.32
6.03
8.04
9.52
11.10
12.01
13.02
16.42
18.03
17.12
21.03
23.28
24.02
25.53
27.01
28.99
30.21
33.04
36.03
39.02
42.02
43.93
45.61
45.68
48.03
48.81
49.21
50.08
51.03
51.36
52.91

B

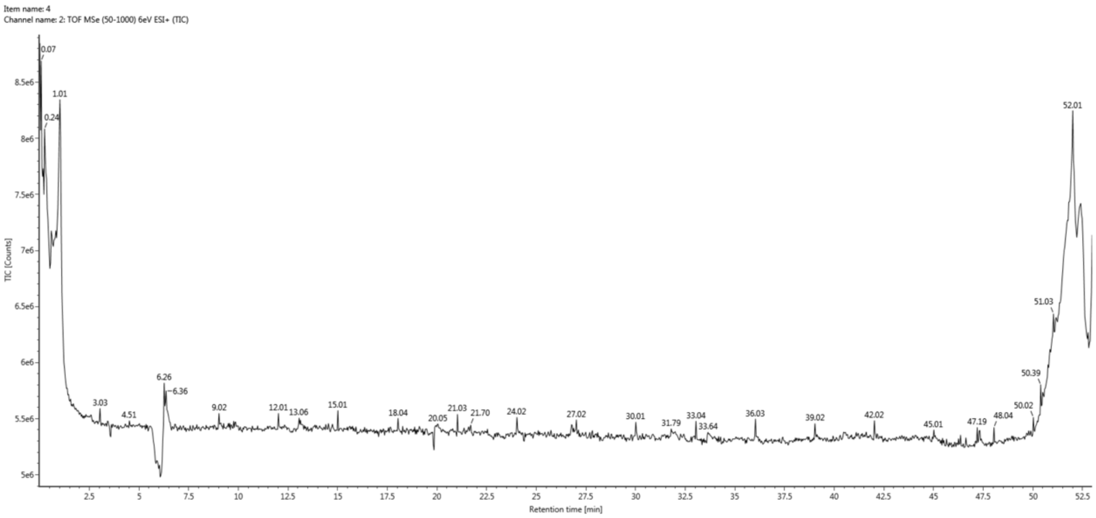
Item name: 4
Channel name: 2: TOF MSe (50-1000) 6eV ESI+ (TIC)
TIC [Counts]
Retention time [min]
0.07
0.24
1.01
3.03
4.51
6.26
6.36
9.02
12.01
13.06
15.01
18.04
20.05
21.03
21.70
24.02
27.02
30.01
31.79
33.04
33.64
36.03
39.02
42.02
45.01
47.19
48.04
50.02
50.39
51.03
52.01

C

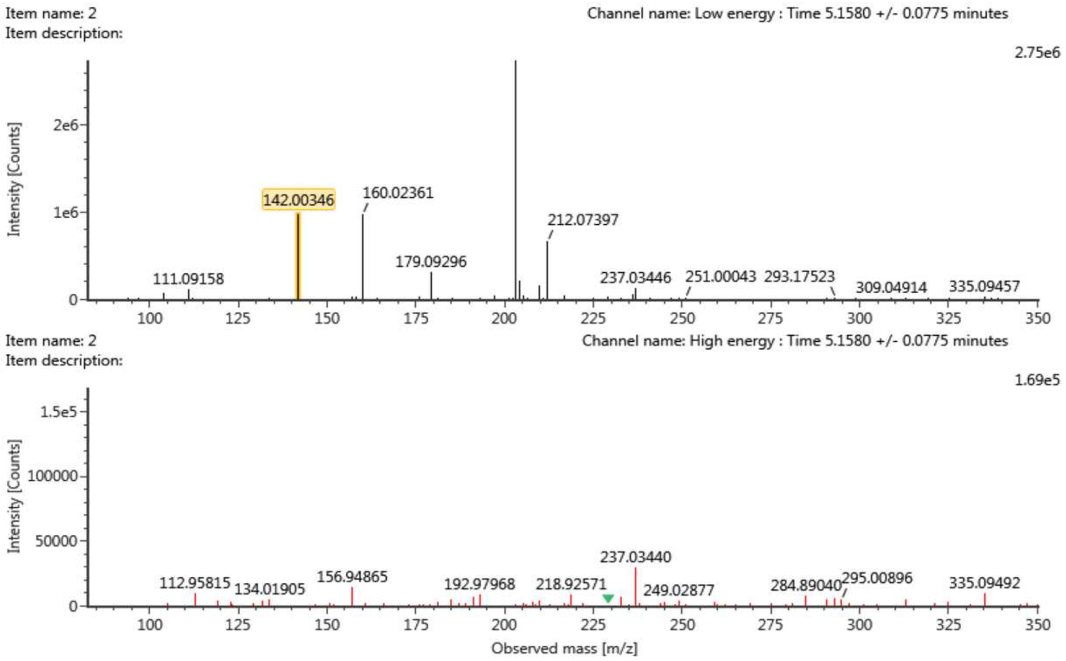
Item name: 2
Item description:
Channel name: Low energy : Time 5.1580 +/- 0.0775 minutes
2.75e6
Intensity [Counts]
142.00346
160.02361
212.07397
179.09296
111.09158
237.03446
251.00043
293.17523
309.04914
335.09457
Item name: 2
Item description:
Channel name: High energy : Time 5.1580 +/- 0.0775 minutes
1.69e5
237.03440
112.95815
134.01905
156.94865
192.97968
218.92571
249.02877
284.89040
295.00896
335.09492
Observed mass [m/z]

D

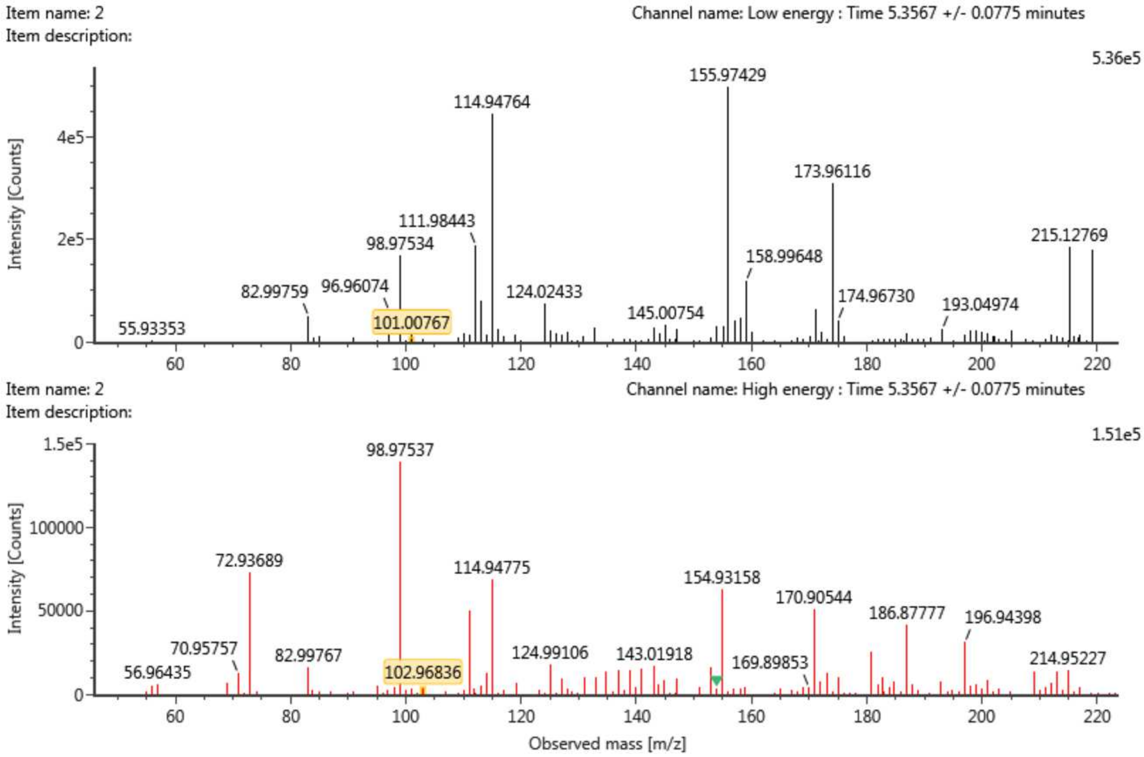
Item name: 2
Item description:
Channel name: Low energy : Time 5.3567 +/- 0.0775 minutes
5.36e5
Intensity [Counts]
155.97429
114.94764
173.96116
111.98443
98.97534
215.12769
158.99648
82.99759
96.96074
124.02433
174.96730
193.04974
145.00754
101.00767
55.93353
Item name: 2
Item description:
Channel name: High energy : Time 5.3567 +/- 0.0775 minutes
1.51e5
98.97537
72.93689
114.94775
154.93158
170.90544
186.87777
196.94398
70.95757
82.99767
124.99106
143.01918
169.89853
214.95227
56.96435
102.96836
Observed mass [m/z]

E

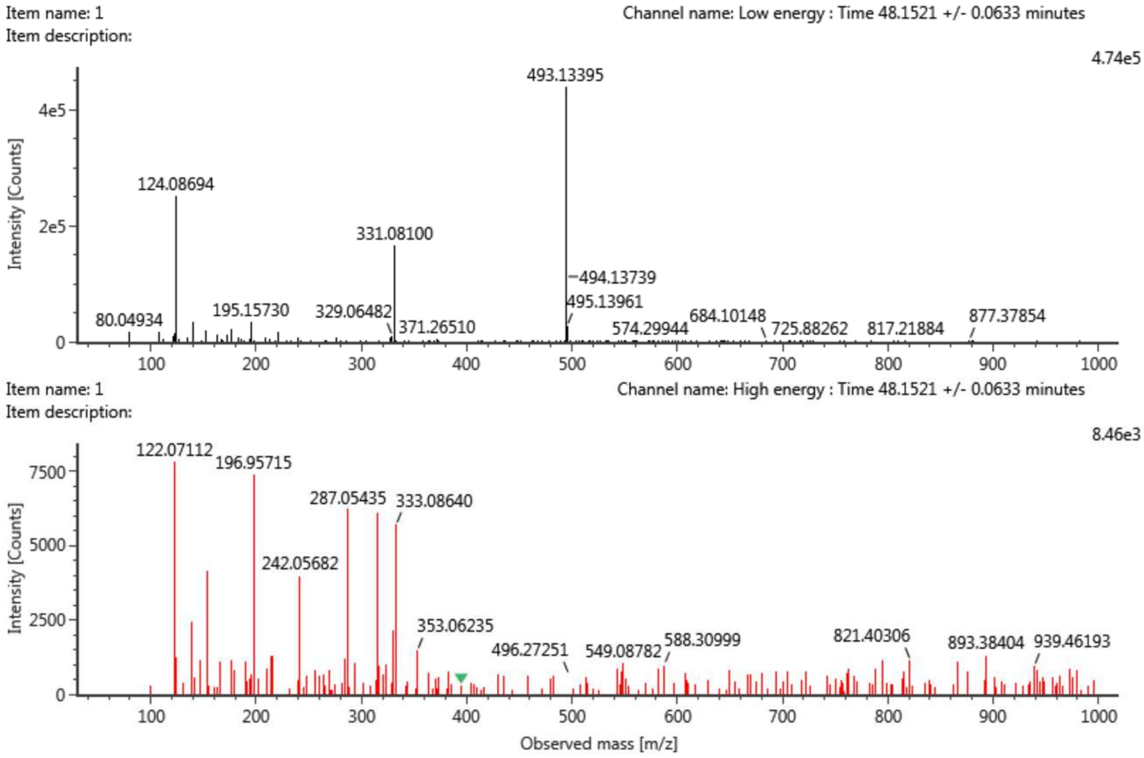
Item name: 1
Item description:
Channel name: Low energy : Time 48.1521 +/- 0.0633 minutes
4.74e5
Intensity [Counts]
493.13395
124.08694
331.08100
494.13739
495.13961
80.04934
195.15730
329.06482
371.26510
574.29944
684.10148
725.88262
817.21884
877.37854
Item name: 1
Item description:
Channel name: High energy : Time 48.1521 +/- 0.0633 minutes
8.46e3
122.07112
196.95715
287.05435
333.08640
242.05682
353.06235
496.27251
549.08782
588.30999
821.40306
893.38404
939.46193
Observed mass [m/z]

F

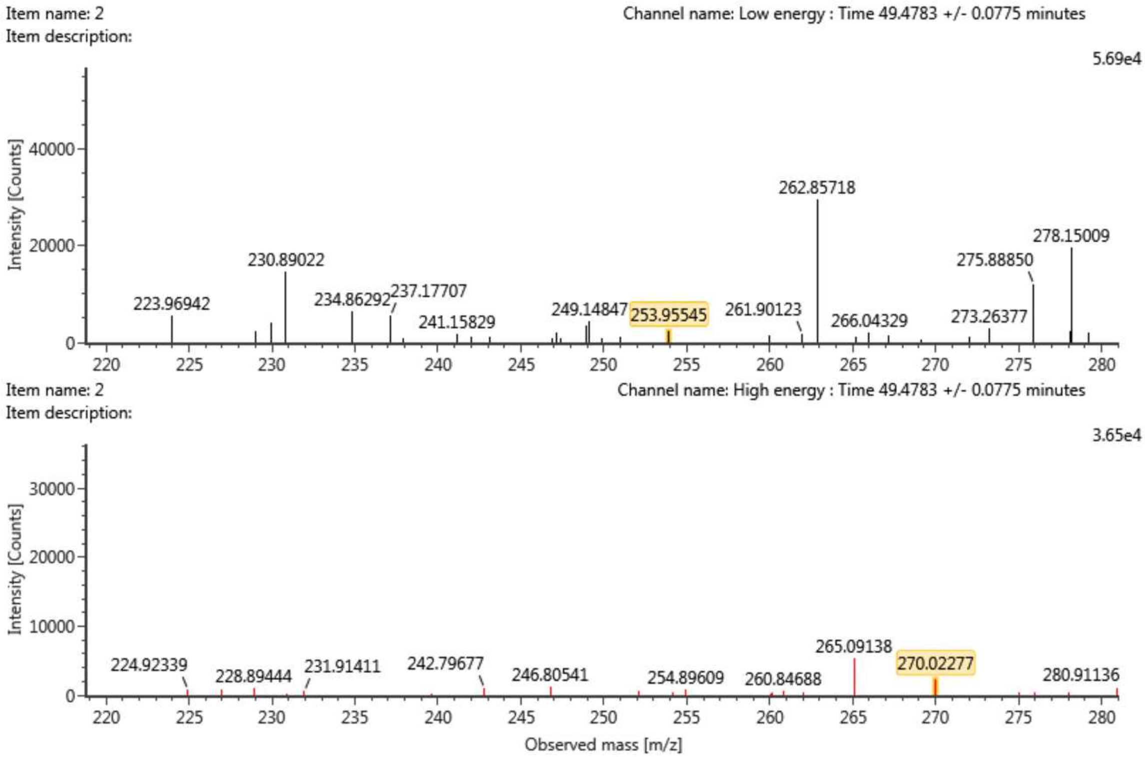
Item name: 2
Item description:
Channel name: Low energy : Time 49.4783 +/- 0.0775 minutes
5.69e4
Intensity [Counts]
262.85718
278.15009
275.88850
230.89022
223.96942
234.86292
237.17707
241.15829
249.14847
253.95545
261.90123
266.04329
273.26377
Item name: 2
Item description:
Channel name: High energy : Time 49.4783 +/- 0.0775 minutes
3.65e4
265.09138
270.02277
224.92339
228.89444
231.91411
242.79677
246.80541
254.89609
260.84688
280.91136
Observed mass [m/z]

G

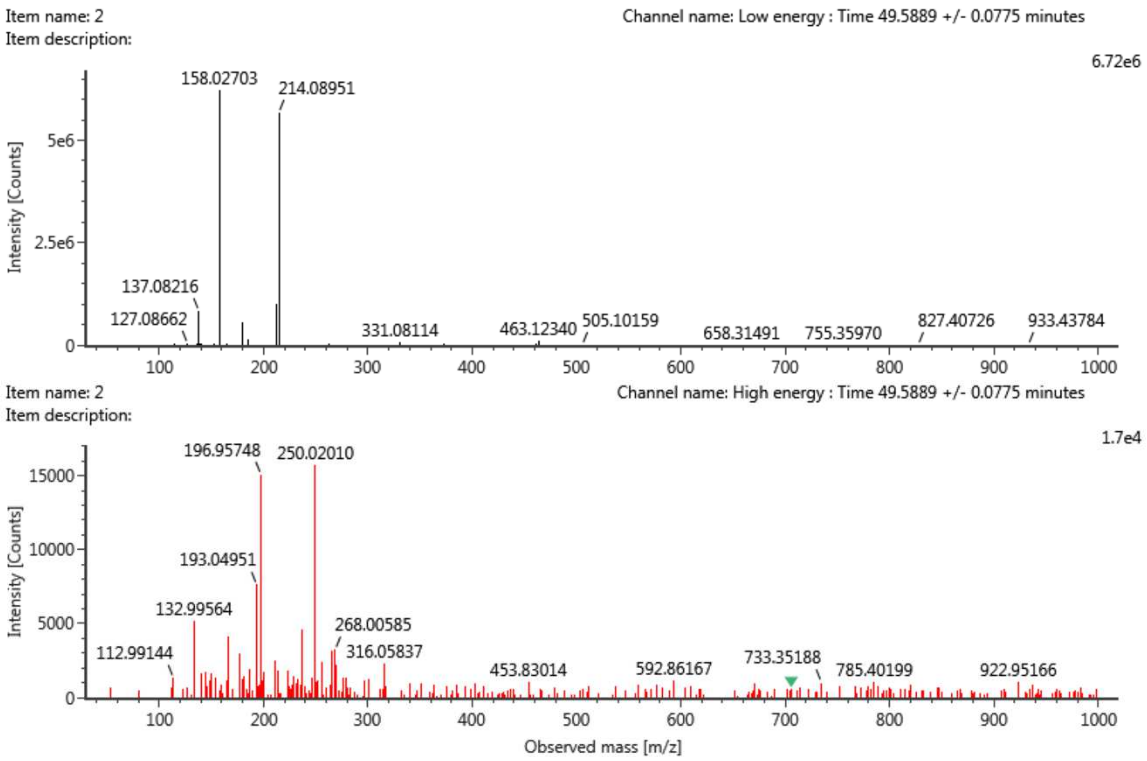
Item name: 2
Item description:
Channel name: Low energy : Time 49.5889 +/- 0.0775 minutes
6.72e6
Intensity [Counts]
158.02703
214.08951
137.08216
127.08662
331.08114
463.12340
505.10159
658.31491
755.35970
827.40726
933.43784
Item name: 2
Item description:
Channel name: High energy : Time 49.5889 +/- 0.0775 minutes
1.7e4
196.95748
250.02010
193.04951
132.99564
268.00585
112.99144
316.05837
453.83014
592.86167
733.35188
785.40199
922.95166
Observed mass [m/z]

H

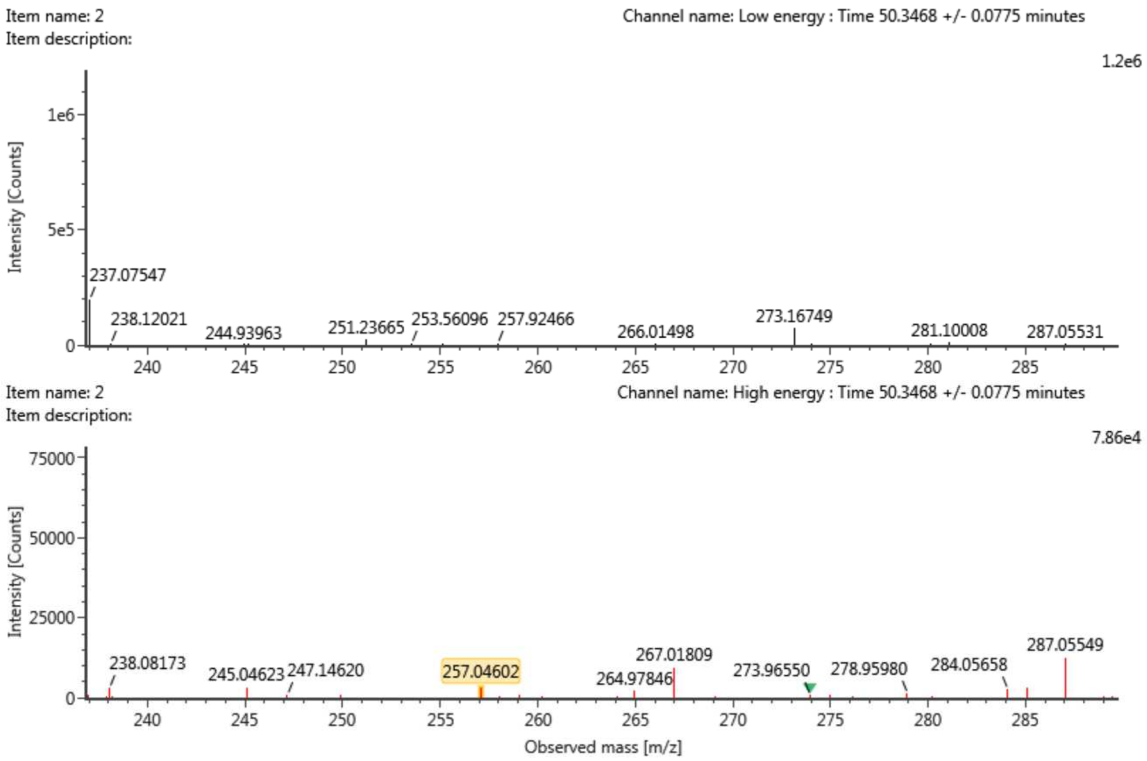
Item name: 2
Item description:
Channel name: Low energy : Time 50.3468 +/- 0.0775 minutes
1.2e6
Intensity [Counts]
237.07547
238.12021
244.93963
251.23665
253.56096
257.92466
266.01498
273.16749
281.10008
287.05531
Item name: 2
Item description:
Channel name: High energy : Time 50.3468 +/- 0.0775 minutes
7.86e4
238.08173
245.04623
247.14620
257.04602
264.97846
267.01809
273.96550
278.95980
284.05658
287.05549
Observed mass [m/z]

I

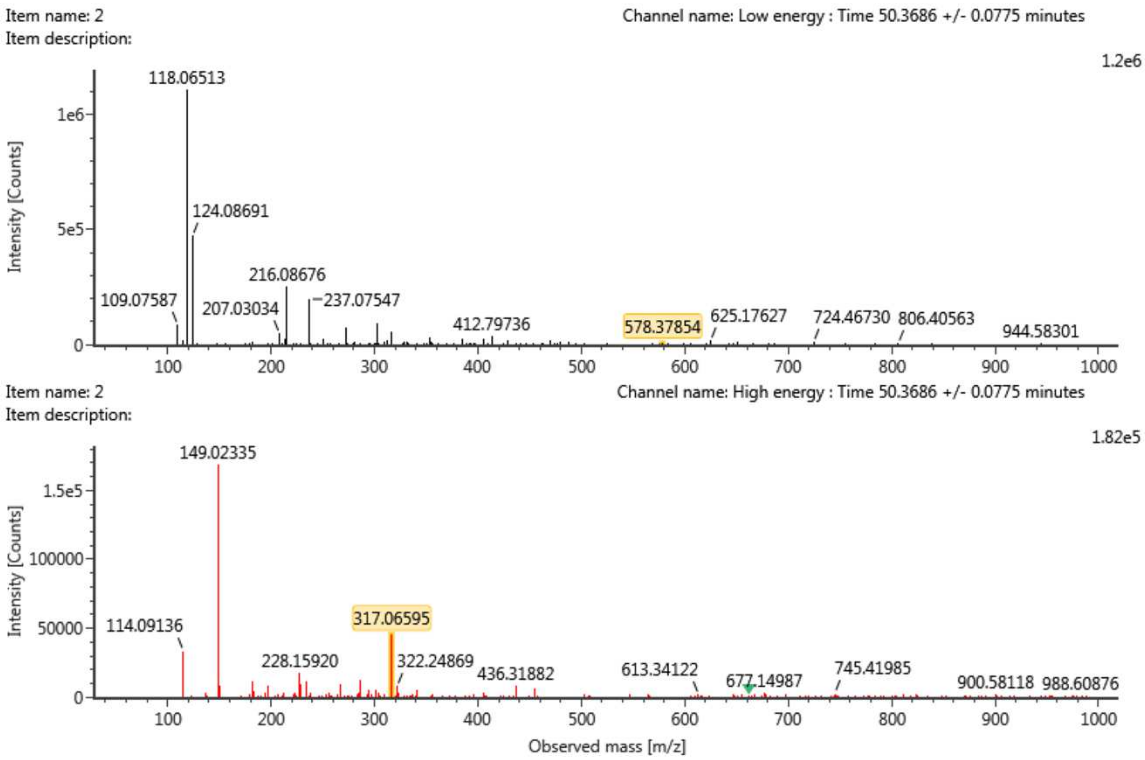

Item name: 2
Item description:
Channel name: Low energy : Time 50.3686 +/- 0.0775 minutes
1.2e6
118.06513
124.08691
216.08676
109.07587
207.03034
237.07547
412.79736
578.37854
625.17627
724.46730
806.40563
944.58301
Intensity [Counts]
Item name: 2
Item description:
Channel name: High energy : Time 50.3686 +/- 0.0775 minutes
1.82e5
149.02335
114.09136
317.06595
228.15920
322.24869
436.31882
613.34122
677.14987
745.41985
900.58118
988.60876
Observed mass [m/z]

J

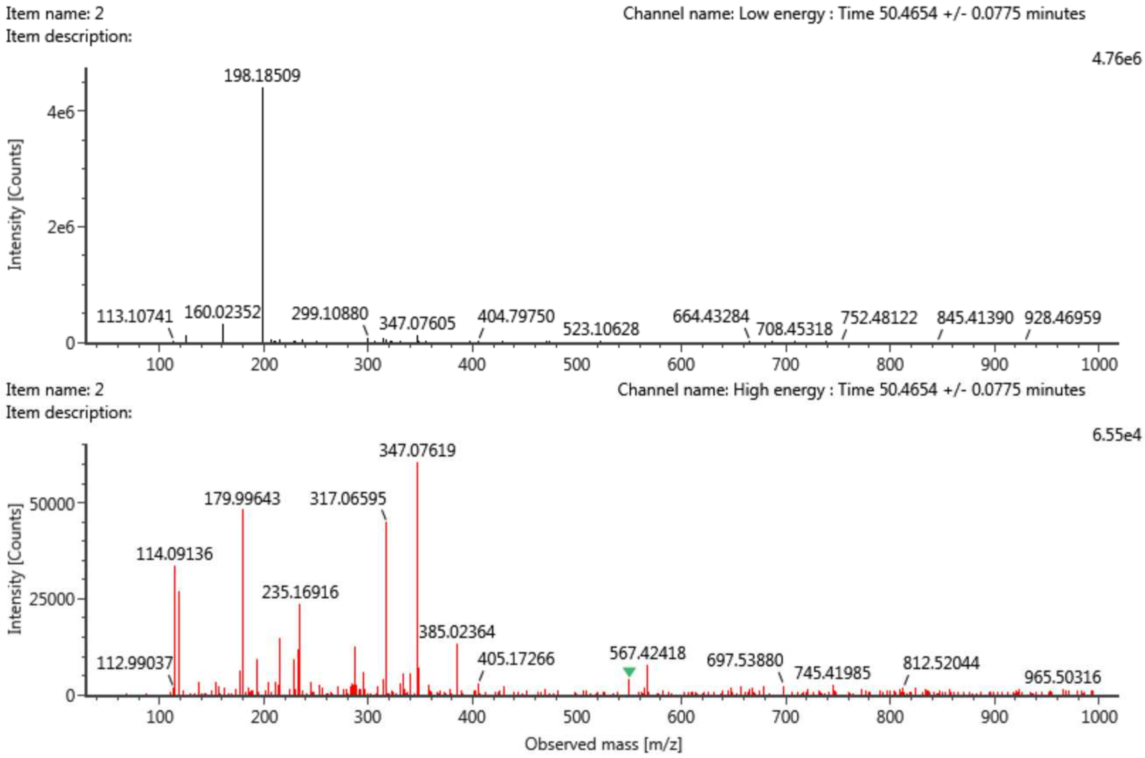

Item name: 2
Item description:
Channel name: Low energy : Time 50.4654 +/- 0.0775 minutes
4.76e6
198.18509
113.10741
160.02352
299.10880
347.07605
404.79750
523.10628
664.43284
708.45318
752.48122
845.41390
928.46959
Intensity [Counts]
Item name: 2
Item description:
Channel name: High energy : Time 50.4654 +/- 0.0775 minutes
6.55e4
347.07619
179.99643
317.06595
114.09136
235.16916
385.02364
112.99037
405.17266
567.42418
697.53880
745.41985
812.52044
965.50316
Observed mass [m/z]

K

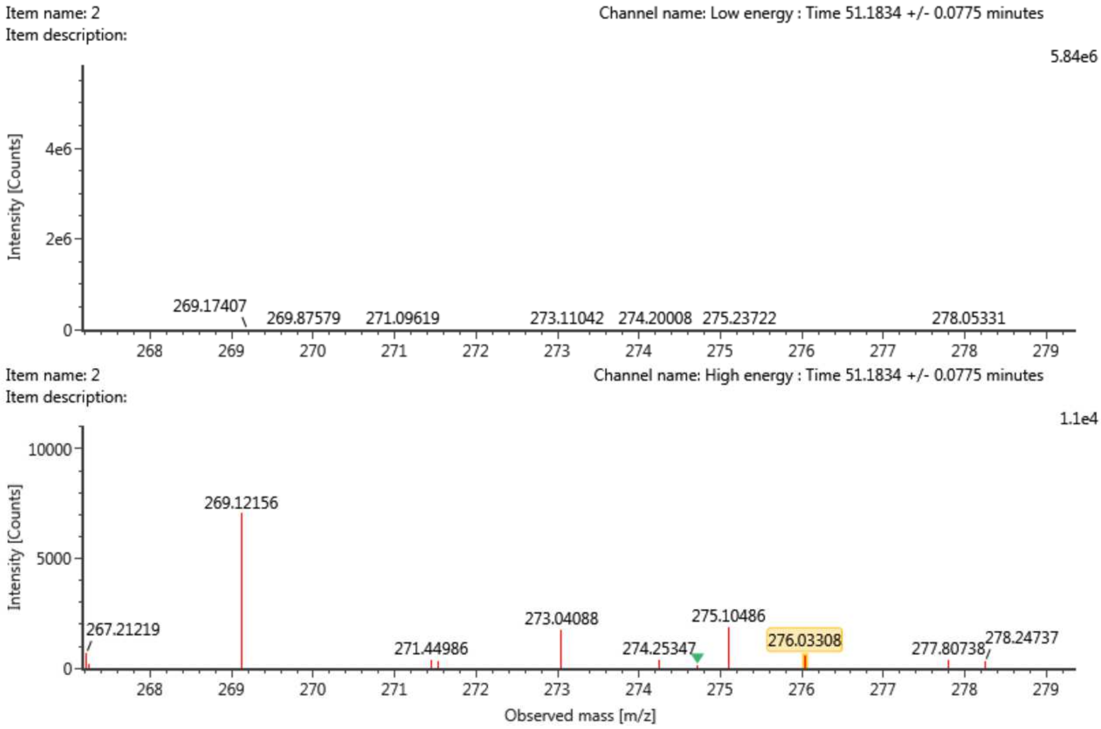

Figure S1. (A) The total ion chromatogram of centrifuged blueberry juice (before SEC). (B) The total ion chromatogram of B-EVs. Mass spectrogram of 3-O-Methylgallic acid (C), Coumarin (D), Malvidin 3-O-β-D-galactoside (E), Isopeonidin 3-O-arabinoside (F), Malvidin 3-arabinosid (G), 5,6,7,3′,4′-Pentahydroxyisoflavone (H), Petunidin- glucoside (I), Luteolin (J), 6-O-Malonylgenistin (K).
